# Biosynthesis and Translocation of Unsulfated Acyltrehaloses in Mycobacterium tuberculosis*

**DOI:** 10.1074/jbc.M114.581199

**Published:** 2014-08-14

**Authors:** Juan Manuel Belardinelli, Gérald Larrouy-Maumus, Victoria Jones, Luiz Pedro Sorio de Carvalho, Michael R. McNeil, Mary Jackson

**Affiliations:** From the ‡ Mycobacteria Research Laboratories, Department of Microbiology, Immunology and Pathology, Colorado State University, Fort Collins, Colorado 80523-1682 and; the § Division of Mycobacterial Research, Medical Research Council National Institute for Medical Research, London NW7 1AA, United Kingdom

**Keywords:** Glycolipid, Mycobacteria, Polyketide, Transporter, Tuberculosis, Chp2, MmpL10, Acyltransferase, Acyltrehalose

## Abstract

A number of species-specific polymethyl-branched fatty acid-containing trehalose esters populate the outer membrane of *Mycobacterium tuberculosis*. Among them, 2,3-diacyltrehaloses (DAT) and penta-acyltrehaloses (PAT) not only play a structural role in the cell envelope but also contribute to the ability of *M. tuberculosis* to multiply and persist in the infected host, promoting the intracellular survival of the bacterium and modulating host immune responses. The nature of the machinery, topology, and sequential order of the reactions leading to the biosynthesis, assembly, and export of these complex glycolipids to the cell surface are the object of the present study. Our genetic and biochemical evidence corroborates a model wherein the biosynthesis and translocation of DAT and PAT to the periplasmic space are coupled and topologically split across the plasma membrane. The formation of DAT occurs on the cytosolic face of the plasma membrane through the action of PapA3, FadD21, and Pks3/4; that of PAT occurs on the periplasmic face via transesterification reactions between DAT substrates catalyzed by the acyltransferase Chp2 (Rv1184c). The integral membrane transporter MmpL10 is essential for DAT to reach the cell surface, and its presence in the membrane is required for Chp2 to be active. Disruption of *mmpL10* or *chp2* leads to an important build-up of DAT inside the cells and to the formation of a novel form of unsulfated acyltrehalose esterified with polymethyl-branched fatty acids normally found in sulfolipids that is translocated to the cell surface.

## Teaser

**Background:** The biogenesis of 2,3-diacyltrehaloses (DAT) and penta-acyltrehaloses (PAT) found in the outer membrane of *Mycobacterium tuberculosis* is ill defined.

**Results:** DAT synthesis is cytosolic. Chp2-mediated transesterification reactions between DAT substrates yield PAT on the periplasmic face of the membrane.

**Conclusion:** DAT and PAT biosynthesis is topologically split across the membrane.

**Significance:** DAT and PAT biosynthesis and transport are coupled and dependent on the MmpL10 transporter.

## Introduction

It is now generally accepted that the organisms belonging to the corynebacteria-mycobacteria-nocardia group possess a complex cell envelope made of an inner plasma membrane, the peptidoglycan-arabinogalactan complex, and a pseudo-outer membrane also referred to as “mycomembrane” (1–5). In the interest of simplicity, the term “outer membrane” will here be used to designate the mycomembrane, and “periplasm” will be used to define the space located between the mycobacterial inner and outer membranes.

**FIGURE 1.**
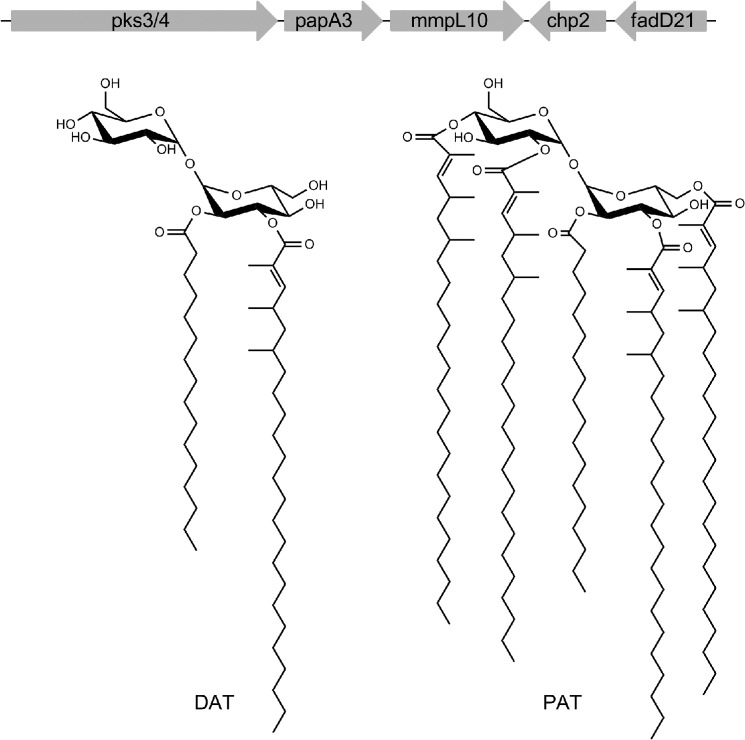
**DAT and PAT structures and biosynthetic gene cluster.** Genes associated with DAT and PAT biosynthesis and export are clustered on the *M. tuberculosis* H37Rv chromosome. In the forms of DAT and PAT represented here, trehalose is esterified with palmitic acid and multimethyl-branched mycolipenic acids.

Lipids and glycolipids of unusual structures populate the outer membrane of mycobacteria, governing various aspects of their physiology and pathogenicity (1–2, 5). Among them, the acyltrehaloses produced by *Mycobacterium tuberculosis*, which include sulfolipids (SL),^2^ diacyltrehaloses (DAT), and penta- (or poly-) acyltrehaloses (PAT), have in common a basic structure consisting of a trehalose moiety esterified with one middle-chain saturated fatty acid (palmitic or stearic acid) at the 2-position and up to four polymethyl-branched long-chain fatty acids at the 3-, 6-, 2′-, 4′-, or 6′-position (Fig. 1). The polymethyl-branched fatty acids found in SL are known as the (C31–C46) phthioceranic and hydroxyphthioceranic acids, whereas (C21–C28) mycosanoic, mycolipenic, and mycolipanolic acids esterify trehalose in the case of DAT and PAT. In addition, the trehalose moiety of SL is sulfated at the 2′-position, a modification not found in the unsulfated DAT and PAT. DAT and PAT are unique to *M. tuberculosis* complex species, and SL are exclusively found in the human pathogen, *M. tuberculosis*.

*In vitro* studies using purified SL, DAT, and PAT indicate that they are biologically active molecules capable of modulating a number of cell functions and host immune responses (6–18). *M. tuberculosis* knock-out mutants deficient in their synthesis, however, failed to show any consistent virulence phenotype in animal and cellular models of tuberculosis infection (18–27) unless simultaneously impaired in their ability to synthesize phthiocerol dimycocerosates (PDIM) (28), suggestive of partially redundant functions among polymethyl-branched fatty acid-containing lipids. Recent evidence indicates that one of these functions is to alleviate the propionate-mediated stress undergone by the bacilli during growth on host cholesterol as a major carbon source (29–30). The contribution of these lipids to blocking the phagosome acidification of infected macrophages further suggests that their presence at the cell surface may promote the intracellular survival of *M. tuberculosis* (28).

Similarities in the genetic organization of the SL and DAT/PAT biosynthetic gene clusters is suggestive of conserved mechanisms of assembly and export for both families of acyltrehaloses. To this date, however, only two genes of the DAT/PAT biosynthetic cluster have been characterized (Fig. 1). *pks3/4* encodes the polyketide synthase responsible for the elongation of mycosanoic and mycolipenic acids (31), whereas *papA3* encodes the acyltransferase that catalyzes the sequential esterification of the 2- followed by the 3-position of trehalose, leading to the formation of DAT (32). Disruption of *pks3/4* and *papA3* in *M. tuberculosis* yields mutants devoid of DAT and PAT. By analogy to the better studied SL biosynthetic pathway (2, 33), we hypothesized that FadD21 is the fatty acid AMP ligase that provides the activated fatty acid starter unit to Pks3/4, whereas Chp2 (Rv1184c) transfers the remaining three mycolipenoyl groups onto DAT to form PAT, and MmpL10 is an inner membrane RND (resistance, nodulation, and division) transporter required for the translocation of DAT and/or PAT to the cell surface. This work was undertaken with the goals of not only establishing the involvement of FadD21, Chp2, and MmpL10 in DAT and PAT biosynthesis but also addressing a number of outstanding questions related to the assembly and export of these lipids including: (i) the sequential order of the reactions leading to the synthesis and export of DAT and PAT; (ii) the topology of the pathway; (iii) the determination of whether a multiprotein complex coupling biosynthesis and export may be involved; and (iv) the requirement of MmpL10 for the translocation of DAT, PAT, or both substrates to the cell surface. Our results corroborate a model wherein the biosynthesis and translocation of DAT and PAT are coupled and topologically split across the plasma membrane, with the formation of DAT occurring on the cytosolic side of the plasma membrane and that of PAT occurring on the periplasmic face via Chp2-mediated transesterification reactions between DAT substrates.

## EXPERIMENTAL PROCEDURES

### 

#### 

##### Bacterial Strains and Growth Conditions

*M. tuberculosis* mc^2^6206 (an avirulent Δ*panCD*Δ*leuCD* mutant of *M. tuberculosis* H37Rv; kind gift from Dr. W. R. Jacobs Jr., Albert Einstein College of Medicine, New York) and *Mycobacterium smegmatis* mc^2^155 were grown in Middlebrook 7H9 broth with 10% oleic acid-albumin-dextrose-catalase supplement (BD Biosciences), 0.5% glycerol, and 0.05% Tween 80 or on Middlebrook 7H11 agar supplemented with 10% oleic acid-albumin-dextrose-catalase (BD Biosciences) and 0.5% glycerol. All media used to grow *M. tuberculosis* mc^2^6206 were supplemented with 0.2% casamino acids, 48 μg/ml pantothenate, and 20 μg/ml l-leucine. *Escherichia coli* DH5α, the strain used for cloning, was grown in Luria-Bertani (LB) broth or agar (BD Biosciences). Kanamycin (20–50 μg/ml), hygromycin (50–150 μg/ml), ampicillin (100 μg/ml), and 2% sucrose were added to the culture media when needed.

##### Construction of M. tuberculosis Mutants and Complemented Mutant Strains

The construction of *fadD21*, *mmpL10*, and *chp2* (*Rv1184c*) deletion mutants of *M. tuberculosis* mc^2^6206 involved replacing the corresponding entire ORFs by the kanamycin resistance cassette from pUC4K (GE Healthcare) following standard allelic replacement strategies with pPR27-xylE, a replicative plasmid harboring a temperature-sensitive origin of replication, the counterselectable marker *sacB*, and the colored marker *xylE* (34). Details of the plasmid constructs are available upon request. Complementation constructs for *fadD21* and *chp2* consist of the full-size genes, PCR-amplified from *M. tuberculosis* mc^2^6206 genomic DNA and expressed under control of the p*hsp*60 promoter from the replicative plasmid pMVGH1 (35). The complementation construct used in the case of the *mmpL10* mutant, pNIP40b-*mmpL10*, consists of the *mmpL10* gene expressed from its own promoter in the integrative plasmid pNIP40b (36).

##### Metabolic Labeling

*M. tuberculosis* cultures grown to mid-exponential phase (*A*_600_ = 0.5–0.6) were added 0.5 μCi/ml [1-^14^C]propionate (specific activity, 55 Ci/mol, American Radiolabeled Chemicals, Inc.) and labeled for 24 h at 37 °C with shaking. Cell pellets were washed twice with phosphate-buffered saline prior to lipid extraction.

##### Lipid Extraction and Analyses

Surface-exposed lipids extracted with water-saturated butanol and cell pellet-associated lipids extracted with chloroform and methanol were analyzed by one- and two-dimensional thin layer chromatography (TLC) following procedures described earlier (35, 37). Radiolabeled products were visualized using a PhosphorImager (Typhoon, GE Healthcare). SL-1, DAT, PAT, and lipid AT-X were purified by preparative TLC or as described elsewhere (32, 38). Total, acetone-soluble, and purified lipids were suspended in chloroform/methanol (8:2, v/v) and directly deposited onto a steel target for analysis by MALDI-TOF MS (microflex LRF (Bruker Daltonics, Billerica, MA) or Ultraflex (Bruker, Bremen, Germany)). Spectra were acquired in reflectron mode and mass-assigned through external calibration. 2-(4-Hydroxyphenylazo)benzoic acid or 2,5-dihydroxybenzoic acid matrix (Sigma) was used at a concentration of ∼10 mg/ml in ethanol/water (1:1, v/v). In a typical experiment, 1 μl of glycolipid (5–10 μg) in chloroform/methanol (8:2, v/v) and 1 μl of the matrix solution were mixed with a micropipette directly on the target. MALDI-TOF/MS/MS was performed using the LIFT method. One-dimensional ^1^H and two-dimensional ^1^H-^1^H COSY and ^1^H-^13^C HSQC NMR spectroscopy were carried out on a Bruker 600- and 800-MHz NMR spectrometer, equipped with a 5-mm triple resonance probe head and *z* axis pulsed field gradients. AT-X was dissolved in CDCl_3_-CD_3_OD (8:2, v/v) and analyzed in 200 5-mm 535 PP NMR tubes at 295 K. Proton chemical shifts are expressed in ppm downfield from the signal of chloroform (δ_H_/TMS 7.26 and δ_C_/TMS 77.7).

##### GC/MS Analysis of Fatty Acyl Groups

The glycolipid AT-X was treated with 3 m HCl in CH_3_OH (Supelco) overnight at 80 °C to both release the fatty acyl groups from AT-X and form their methyl esters. The sample was then dried and dissolved in 50 μl of *N*,*O*-bis(trimethylsilyl) trifluoroacetamide (Sigma-Aldrich) and heated at 60 °C for 10 min prior to injection for GC/MS to form the trimethylsilyl ethers of any hydroxyl groups. Samples were injected directly from the silylating reagent. Analyses were carried out using a CP 3800 gas chromatograph (Varian) equipped with an MS320 mass spectrometer in the electron impact mode and scanning from *m*/*z* 50 to 800 over 0.5 s. Helium was used as the carrier gas with a flow rate of 1 ml/min. The samples were run on a DB 5 column (10 m × 0.20-mm inner diameter). The injector (splitless mode) was set for 250 °C. The oven temperature was held at 50 °C for 1 min, programmed at 30 °C/min to 130 °C, and then programmed at 10 °C/min to 330 °C, followed by a 10-min hold. The data analyses were carried out on a Varian WS data station.

##### Topology of Chp2 in E. coli and M. smegmatis

A gene fusion approach combining the alkaline phosphatase gene (*phoA*) and the α-fragment of the β-galactosidase (*lacZ*α) was used to establish the topology of the catalytic domains of Chp2 in *E. coli*. To this end, the full-length *chp2* gene fused at its 3′ end in frame with the dual *phoA-lacZ*α reporter cassette from pMA632 (39) was inserted at the HindIII site of pUC19, yielding pUC-[chp2-phoA-lacZ]. Control plasmids harbored either no insert or the only *phoA-lacZ*α reporter cassette expressed from the *lacZ* promoter of pUC19. *E. coli* DH5α transformed with these plasmids was plated on dual indicator plates containing 80 μg/ml 5-bromo-4-chloro-3-indolyl phosphate, 100 μg/ml 6-chloro-3-indolyl-β-d-galactoside (Red-Gal), 1 mm isopropyl 1-thio-β-d-galactopyranoside, and 80 mm K_2_HPO_4_ (pH 7.0) to assess concomitantly PhoA and β-galactosidase activities (39).

To establish the subcellular localization of the catalytic sites of Chp2 and Chp1 in mycobacteria, the mycobacterial expression plasmids pJB(−) and JB(+) were engineered in-house from pMV261 (40) and the *E. coli* expression plasmids pWARF(+) and pWARF (−) (41) to allow for the mycobacterial expression of proteins C-terminally fused to the green fluorescent protein (GFP).^3^ Briefly, in pJB(+), a single transmembrane domain from glycophorin A is added between the C-terminal fusion point of the protein of interest and the GFP to convert membrane proteins with extracellular C-terminal fusions to proteins with intracellular C-terminal fusions. Because GFP fluoresces in the cytoplasm but not in the periplasm, a high fluorescence signal in the pJB(−) version and background fluorescence in the pJB(+) version are indicative of the C-terminal fusion of the protein being cytoplasmic. Opposite fluorescence intensities indicate, on the contrary, that the C-terminal fusion of the protein is localized in the periplasm (41). Fusions between the C-terminal ends of Chp2 or Chp1 and GFP were generated in pJB(−) and pJB(+) and used to transform *M. smegmatis*. Control pJB(−) and pJB(+) plasmids harbor GFP fusions with the C-terminal ends of EmbC and PimA. EmbC is a decaprenyl phosphate arabinose-dependent arabinosyltransferase whose C-terminal domain is periplasmic (42–43). PimA is a cytoplasmic GDP-mannose-dependent mannosyltransferase (44). Cultures of transformants grown to log phase, washed twice with PBS, and resuspended in 100 μl of the same buffer were transferred to black 96-well plates with transparent bottoms (Corning, Inc.), and their fluorescence was determined using a 2030 MultiLabel Reader Victor X5 plate reader (PerkinElmer Life Sciences) at excitation and emission wavelengths of 485 and 535 nm, respectively. The fluorescence value of each sample was normalized to the *A*_600_ of the culture.

##### Biochemical Characterization of Chp2

The Chp2 protein devoid of its N-terminal transmembrane domain was produced in *E. coli*. To this end, the *chp2* gene was amplified from genomic *M. tuberculosis* H37Rv genomic DNA by standard PCR using primers Chp2Fw (5′-AAGCCATATGGCGTACCCGTGGGCTCCTGGG-3′) and Chp2Rv (5′-TTTGCTCGAGGGCAGTCGATCGTACGCTAGTTA-3′), digested with NdeI and XhoI, and cloned into the expression plasmid pET14b (Novagen), yielding pET14b-*chp2*. Following a 3–4-h induction with 1 mm isopropyl 1-thio-β-d-galactopyranoside at 37 °C in LB-ampicillin broth, *E. coli* BL21(DE3) cells transformed with pET14b-*chp2* were harvested, washed, and resuspended in lysis buffer consisting of 100 mm potassium phosphate (pH 7.2) and 5 mm imidazole. Cells were disrupted by sonication and the clarified lysate was incubated with HIS-Select® High Flow (HF) nickel affinity gel (Sigma) for 1 h at 4 °C. The gel was then washed six times with lysis buffer, and the protein was eluted with an increasing gradient of 25–250 mm imidazole in lysis buffer. The elution fractions were concentrated using a Vivaspin® 6 centrifugal device (Viva Products) prior to use in enzyme assays.

##### In Vitro PAT Synthesis

The assay used to analyze the activity of the catalytic domain of Chp2 *in vitro* consisted of incubating ^14^C-labeled DAT (∼2000 cpm) with 15 μg of purified Chp2 catalytic domain in 1 ml of reaction buffer (100 mm potassium phosphate (pH 7.2) and 1 mm DTT). The lipase inhibitor tetrahydrolipstatin (THL) (40 μg/ml) was added to some reaction mixtures. ^14^C-Labeled DAT was purified by preparative TLC from the [1-^14^C]propionate-derived lipids of the *M. tuberculosis mmpL10* knock-out mutant. Reaction mixtures were incubated overnight at room temperature, and the products of the reactions extracted with chloroform/methanol (33) were analyzed by TLC. Assays with [^14^C]C16:0 (10 μm; 55 mCi/mmol; American Radiochemicals Inc.) and 10 μm CoA used whole cell lysates prepared from the *E. coli* control and *chp2*-expressing strains (600 μg of total proteins) to generate [^14^C]C16:0-CoA *in situ* and non-radiolabeled DAT (0, 1, or 10 μm) as the acceptor substrate. The reactions were performed, and the products of the reaction were analyzed as described above.

## RESULTS

### 

#### 

##### Construction of fadD21, chp2, and mmpL10 Deletion Mutants of M. tuberculosis H37Rv

**FIGURE 2.**
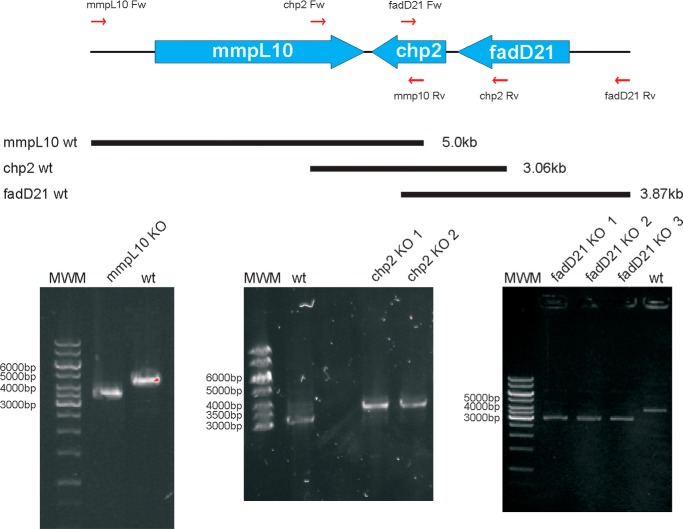
**Disruption of the *fadD21*, *chp2* (*Rv1184c*), and *mmpL10* genes of *M. tuberculosis* H37Rv mc^2^6206.** 1–3 candidate mutants obtained for each of the three genes were analyzed by PCR. The expected sizes of the PCR fragments for the wild-type parent strain are indicated in the schematic representation of the DAT/PAT locus. A 1.2-kb kanamycin-resistance cassette replaces the ORFs in each of the knock-out mutants. Thus, sizes are 3.87 kb for the wild-type parent strain and 3.12 kb for the knock-out mutants in the case of *fadD21*; 3.06 kb for the wild-type parent and 3.44 kb for the knock-out mutants in the case of *chp2*; and 5.0 kb for the wild-type parent and 3.5 kb for the knock-out mutants in the case of *mmpL10. MWM*, molecular weight marker. *wt*, wild-type parent strain.

The involvement of FadD21, Chp2, and MmpL10 in the biosynthesis of DAT and PAT was assessed by generating deletion mutants in the avirulent auxotrophic (Δ*panCD*Δ*leuCD*) *M. tuberculosis* H37Rv strain mc^2^6206 (Fig. 2) and comparing the surface-exposed and intracellular lipid profiles of the mutants with that of their wild-type parent. Complemented mutant strains expressing wild-type copies of *fadD21*, *chp2*, and *mmpL10* from replicative or integrative expression plasmids were generated by transforming the corresponding knock-out mutants with pMVGH1-*fadD21*, pMVGH1-*chp2*, and pNIP40b-*mmpL10*, as described under “Experimental Procedures.” Wild-type, mutant, and complemented mutant strains were metabolically labeled with [1-^14^C]propionate, which preferentially incorporates in the methyl-branched fatty acid-containing lipids (37) to facilitate the detection of biosynthetic intermediates and end products of the SL, DAT, and PAT pathways.

##### Disruption of fadD21 Results in the Loss of DAT and PAT

**FIGURE 3.**
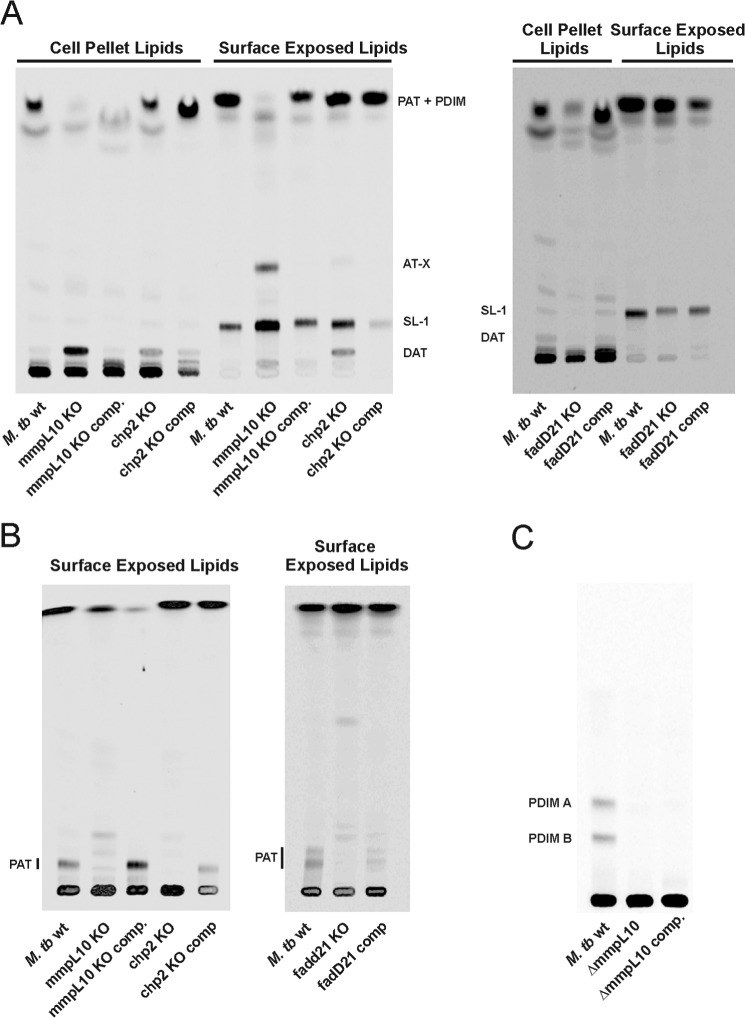
**Effects of knocking out *fadD21*, *chp2*, and *mmpL10* on DAT and PAT biosynthesis and export in *M. tuberculosis*.** Thin layer chromatograms of surface-exposed and cell pellet-associated lipids derived from [1-^14^C]propionate-labeled wild-type, mutant, and complemented mutant strains. 10,000 cpm were loaded per lane. TLC plates were developed in CHCl_3_/CH_3_OH/H_2_O (90:10:1, v/v/v) for DAT, SL-1, and AT-X analysis (*A*) or in petroleum ether/acetone (92:8, v/v) for PAT analysis and revealed by phosphorimaging (*B*). *C*, thin layer chromatogram of total lipids derived from [1-^14^C]propionate-labeled wild-type, *mmpL10* mutant, and complemented *mmpL10* mutant strains showing the absence of PDIM in the mutant and complemented mutant strains. The TLC plate was developed in petroleum ether/diethyl ether (95:5, v/v).

**FIGURE 4.**
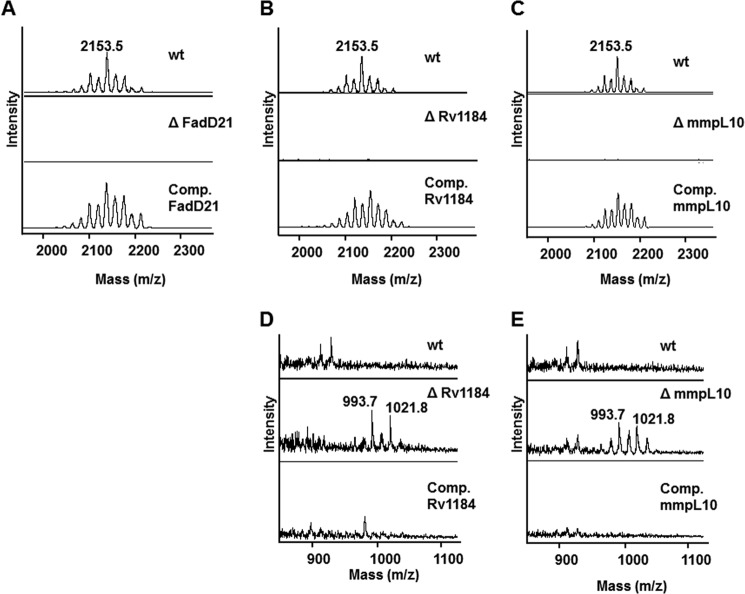
**MALDI-MS analysis of lipids extracted from the wild-type, knock-out mutant, and complemented mutant strains.** Total lipids extracted from wild-type *M. tuberculosis* H37Rv mc^2^6206; the *fadD21*, *chp2 (Rv1184*), and *mmpL10* knock-out mutants; and the complemented mutant strains were precipitated with acetone and subjected to MALDI-MS analysis in the positive ion mode as described under “Experimental Procedures.” Peaks observed are *m*/*z* 2153.5, corresponding to sodium-cationized PAT, and *m*/*z* 993.7 and 1021.8, corresponding to sodium-cationized DAT. DAT accumulate in the cell pellet-associated lipids of the *mmpL10* and *chp2* mutant strains as well as in the surface-exposed lipids of the *chp2* knock-out mutant. No DAT or PAT were detected in the *fadD21* mutant. *A–C* correspond to surface-exposed lipids and focus on the PAT content of the strains; *D* and *E* correspond to cell-associated lipids and focus on the DAT content of the strains.

FadD21 belongs to a family of fatty acyl AMP ligases whose role is to activate long-chain fatty acids as acyl adenylates, which are then transferred to polyketide synthases for further chain extension (45). Consistent with the likely requirement of *fadD21* for the elongation of mycosanoic and mycolipenic acids by Pks3/4, disruption of this gene in *M. tuberculosis* H37Rv resulted in the complete loss of DAT and PAT production that was restored in the complemented mutant strain (Figs. 3 (*A* and *B*) and 4*A*). The identity of the missing lipids was confirmed by MALDI-MS analysis of the corresponding compounds purified by preparative TLC from the wild-type parent strain (data not shown).

##### Involvement of Chp2 in the Biosynthesis of PAT from DAT

The closest homolog of Chp2 is the acyltransferase encoded by *chp1* (*Rv3822*) in the SL biosynthetic cluster (41% sequence identity), which catalyzes the regioselective trans-esterification of two diacylated sulfolipid substrates on the cytosolic face of the plasma membrane to afford SL-I, the final tetraacylated product of the SL biosynthetic pathway (33). Analysis of the surface-exposed and cell pellet-associated lipids produced by the *chp2* null mutant (Fig. 3*B*) revealed an absence of PAT in the mutant strain concomitant with the accumulation of DAT in both lipid fractions (Figs. 3*B* and 4 (*B* and *D*). That the disruption of *chp2* was responsible for this phenotype was supported by the restoration of PAT synthesis in the complemented mutant. The lipid profile of *Mtb*Δ*chp2* is thus suggestive of the involvement of Chp2 in the acylation of DAT with one or more methyl-branched fatty acid products of Pks3/4. Chp2, however, is clearly dispensable for the translocation of DAT to the cell surface.

**FIGURE 5.**
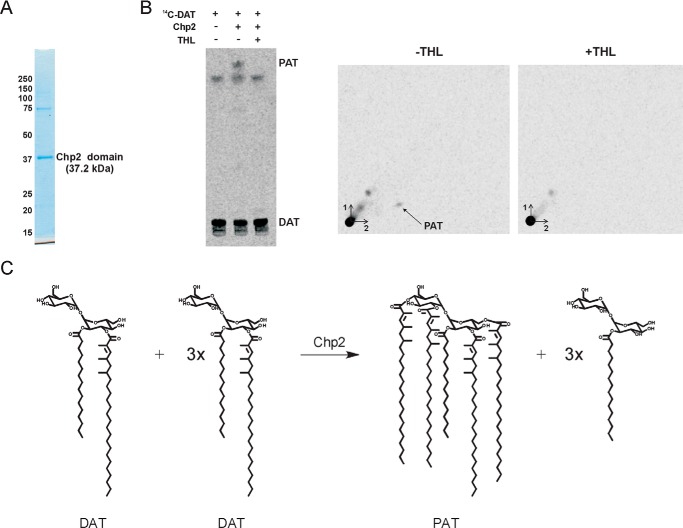
**Chp2 catalyzes the formation of PAT from DAT.** *A*, Coomassie Blue-stained SDS-PAGE showing the recombinant Chp2 protein devoid of N-terminal transmembrane domain purified from *E. coli*; 1.5 μg of protein was loaded on the gel. *B*, 15 μg of purified recombinant Chp2 protein was incubated with ^14^C-labeled DAT (2000 cpm) in the presence or absence of THL (40 μg/ml). The reaction products were analyzed by one- and two-dimensional TLC and revealed by phosphorimaging. One-dimensional TLC plates were developed in CHCl_3_/CH_3_OH/H_2_O (90:10:1, v/v/v). Two-dimensional TLC plates were developed three times in petroleum ether/acetone (92:8, v/v) in the first dimension and once in toluene/acetone (95:5, v/v) in the second dimension. A ^14^C-labeled lipid product with the migration characteristics of PAT is formed when Chp2 is incubated with DAT in the absence of THL. *C*, transesterification reactions between DAT substrates catalyzed by Chp2.

**FIGURE 6.**
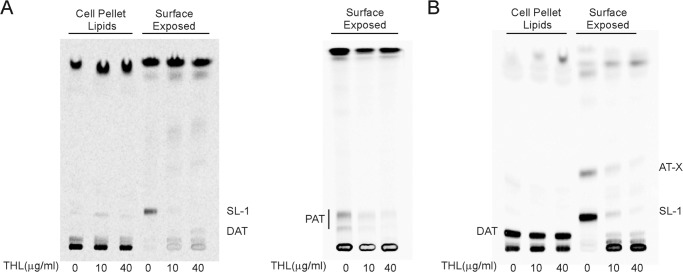
**Inhibition of SL-I, PAT, and AT-X synthesis by the lipase inhibitor THL in whole *M. tuberculosis* cells.** Thin layer chromatograms of surface-exposed and cell pellet-associated lipids derived from [1-^14^C]propionate-labeled wild-type (*A*) and *mmpL10* knock-out mutant (*B*) strains either untreated or treated with THL (10 and 40 μg/ml). The same volume of samples was loaded per lane. TLC plates were developed in CHCl_3_/CH_3_OH/H_2_O (90:10:1, v/v/v) (DAT, SL-1, and AT-X analysis) or three times in petroleum ether/acetone (92:8, v/v) (PAT analysis) and revealed by phosphorimaging.

To gain further insight into the function of Chp2 and determine the number of sequential acylations that this enzyme may catalyze, a recombinant form of Chp2 devoid of the N-terminal transmembrane domain was produced in *E. coli* (Fig. 5*A*), purified, and used in enzyme assays where ^14^C-labeled DAT served both as the donor and acceptor substrates. A ^14^C-labeled lipid product displaying the TLC migration properties of PAT was formed in the reaction mixtures containing both the catalytic domain of Chp2 and ^14^C-labeled DAT (Fig. 5*B*). Attempts to use [^14^C]C16:0-CoA as an acyl donor in similar reactions where cell-free extracts prepared from the same *E. coli* control and *chp2*-expressing strains served as enzyme sources failed to reveal any transfer of [^14^C]C16:0 onto DAT, suggesting that Chp2 is not able to use this acyl donor (data not shown). PAT synthesis *in vitro* was inhibited by the addition of THL to the reaction mixture (Fig. 5*B*), consistent with the partial inhibition of PAT synthesis observed in THL-treated *M. tuberculosis* cells (Fig. 6*A*). The inhibitory effect of THL on PAT synthesis in whole cells (65 and 74% inhibition after 24 h of exposure to 10 and 40 μg/ml of the compound, respectively) was, however, less pronounced than that on SL-I synthesis (96 and 98% inhibition after 24 h of exposure to 10 and 40 μg/ml of the compound, respectively), indicating that THL is a more potent inhibitor of Chp1 (33) than Chp2.

##### The Elaboration of PAT from DAT Occurs on the Periplasmic Face of the Plasma Membrane

**FIGURE 7.**
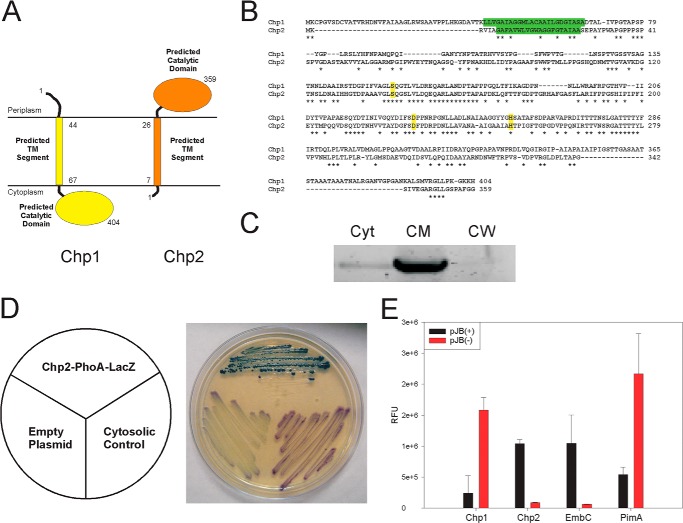
**Subcellular localization and topology of Chp2.** *A*, topology of Chp1 (33) and topology of Chp2 as predicted by HMMTOP version 2.1. *B*, primary sequence alignment of Chp2 and Chp1 showing the N-terminal transmembrane domain (*green highlight*) and putative catalytic triads (Ser-141/Asp-226/His-248 in Chp2) (*yellow highlight*) (33) of these enzymes. Conserved residues are indicated with an *asterisk. C*, subcellular localization of Chp2. Membrane (*CM*), cytosol (*Cyt*), and cell wall (*CW*) fractions were prepared as described (55) from an *M. smegmatis* pJB(−)chp2 transformant expressing a C-terminal GFP-tagged form of Chp2, run on an SDS-polyacrylamide gel (2 μg of protein/lane), and analyzed for the presence of Chp2 by in-gel fluorescence (λ_ex_ = 485 nm, λ_em_ = 525 nm). Chp2 localizes to the cell membrane. *D*, topology of Chp2 in *E. coli*. Plating of *E. coli* DH5α/pUC-[chp2-phoA-lacZ] transformants (expressing *chp2* fused at its C-terminal end to a dual *phoA-lacZ*α reporter cassette) on dual indicator plates containing the substrates for both β-gal and PhoA yielded *blue* colonies indicative of PhoA activity. Transformation of the control plasmid pUC-[phoA-lacZ] yielded the expected *red*/*purple* colonies indicative of β-gal activity. The catalytic site of Chp2 expressed in *E. coli* is thus on the periplasmic side of the plasma membrane. *E*, topology of Chp2 and Chp1 in *M. smegmatis.* The full-length *chp2* and *chp1* genes were fused at their 3′-ends in frame with *gfp* in pJB(−) and JB(+), as described under “Experimental Procedures.” Fluorescence intensities were normalized to the *A*_600_ of the cultures. Fluorescence intensities of *M. smegmatis* pJB(−)chp2, pJB(+)chp2, pJB(−)chp1, and pJB(+)chp1 transformants confirmed the periplasmic location of the catalytic domain of Chp2 and the cytosolic location of the catalytic domain of Chp1. Control pJB(−) and JB(+) plasmids confirmed the periplasmic location of the C-terminal domain of EmbC and the cytosolic location of the C-terminal end of PimA.

Similar to Chp1 (Rv3822), Chp2 is a 359-amino acid-long protein with a single predicted N-terminal transmembrane domain (residues 5–27) and an α/β-hydrolase fold C-terminal domain (residues 28–359) harboring a cutinase-like motif (33) (Fig. 7, *A* and *B*). That Chp2 associates with the membrane was confirmed by expressing a C-terminal GFP-tagged form of this protein in *M. smegmatis* and probing its localization by fluorescence detection upon subcellular fractionation (Fig. 7*C*). To determine whether the catalytic C-terminal domain of Chp2 faced the cytosolic or periplasmic face of the membrane, a construct, pUC-[chp2-phoA-lacZ], was first generated in which the C-terminal end of Chp2 was fused to a dual *phoA-lacZ*α reporter cassette. Because the alkaline phosphatase encoded by *phoA* is only active in the periplasm and the β-galactosidase (β-gal) encoded by *lacZ* is only functional in the cytosol, active PhoA and inactive β-gal indicate a periplasmic location of the fusion, whereas reversed enzyme activities point to a cytoplasmic location of the fusion. Transformation of *E. coli* DH5α with this construct and plating of the transformants on dual indicator plates containing the substrates for both reporter enzymes (Red-Gal and 5-bromo-4-chloro-3-indolyl phosphate; see “Experimental Procedures”) yielded blue colonies indicative of PhoA activity (Fig. 7*D*). Transformation of a control plasmid (pUC-[phoA-lacZ]) in which the *phoA-lacZ*α reporter cassette was directly placed under control of the *lacZ* promoter in pUC19 to allow for the cytosolic production of β-gal yielded, in contrast, the expected red/purple colonies indicative of β-gal activity (Fig. 7*D*). Results thus clearly pointed to the catalytic site of Chp2 being on the periplasmic side of the plasma membrane when expressed in *E. coli*.

To further confirm that the catalytic domain of Chp2 mapped to the periplasmic face of the plasma membrane when expressed in a mycobacterial host, the full-length *chp2* gene was next fused at its 3′-end in frame with *gfp* in pJB(−) and JB(+), yielding plasmids pJB(−)chp2 and pJB(+)chp2 (see “Experimental Procedures”). Because GFP is folded and active only in the cytosol, a high fluorescence signal in *M. smegmatis* pJB (−)chp2 transformants and background fluorescence in pJB (+)chp2 transformants would indicate that the C-terminal catalytic domain of Chp2 is cytoplasmic. Opposite fluorescence intensities would indicate, on the contrary, that this domain is localized in the periplasm (41). Determination of the fluorescence intensities of three independent *M. smegmatis* pJB (−)chp2 and *M. smegmatis* pJB(+)chp2 transformants clearly pointed to the periplasmic location of the catalytic domain of Chp2 (Fig. 7*E*). In-frame C-terminal fusions of the Chp1 protein with GFP in the same plasmids and analysis of the fluorescence intensities of *M. smegmatis* pJB(−)chp1 and pJB(+)chp1 transformants confirmed the cytosolic location of the catalytic domain of this enzyme in mycobacteria (Fig. 7*E*) (33). Control C-terminal GFP fusions of the *M. tuberculosis* EmbC and PimA proteins using the same plasmids confirmed the periplasmic location of the C-terminal end of the first enzyme and the cytosolic location of the C-terminal end of the latter (42–44). It follows that, in contrast to SL biosynthesis wherein the fully acylated SL-I product is elaborated in the cytosol, PAT are elaborated from DAT on the periplasmic face of the plasma membrane.

##### Involvement of mmpL10 in the Biosynthesis of PAT and the Transport of DAT to the Cell Surface

Similar to the situation with *chp2*, deletion of *mmpL10* in *M. tuberculosis* H37Rv mc^2^6206 (Fig. 2) led to a mutant devoid of PAT, which accumulated important amounts of DAT (Figs. 3*A* and 4 (*C* and *E*)). In contrast to the *chp2* mutant, however, DAT only accumulated inside the *mmpL10* mutant cells, and no trace of DAT was found at the cell surface (Figs. 3*A* and 4 (*C* and *E*)). Complementation of *Mtb*Δ*mmpL10* with a wild-type copy of *mmpL10* expressed from pNIP40b-*mmpL10* restored the production of PAT and export of both DAT and PAT in the mutant strain. Thus, MmpL10 is both required for the export of DAT to the cell surface and the formation of PAT in *M. tuberculosis*. Incidentally, in the process of disrupting *mmpL10*, the mutant also lost the ability to produce PDIM (Fig. 2*C*). This loss of PDIM has been shown to occur spontaneously in the process of generating *M. tuberculosis* knock-out mutants (23) and is not related to MmpL10, as evidenced by the absence of PDIM in the complemented mutant strain.

Whether MmpL10 participates in the export of PAT in addition to DAT to the cell surface could not be concluded from these experiments due to the absence of PAT synthesis in the *mmpL10* knock-out mutant. The requirement of MmpL10 for PAT synthesis is reminiscent of the situation described previously in the SL biosynthetic pathway, wherein MmpL8 needs to be present for Chp1 to elaborate the diacylated sulfolipid precursor (SL_1278_) into SL-I (21, 23, 33). A role of MmpL proteins in targeting other enzymes and transporters of the same pathway to the plasma membrane to couple biosynthesis and export was proposed to account for this requirement (33, 46).

##### Evidence of Cross-talk between the SL and DAT/PAT Biosynthetic Pathways

**FIGURE 8.**
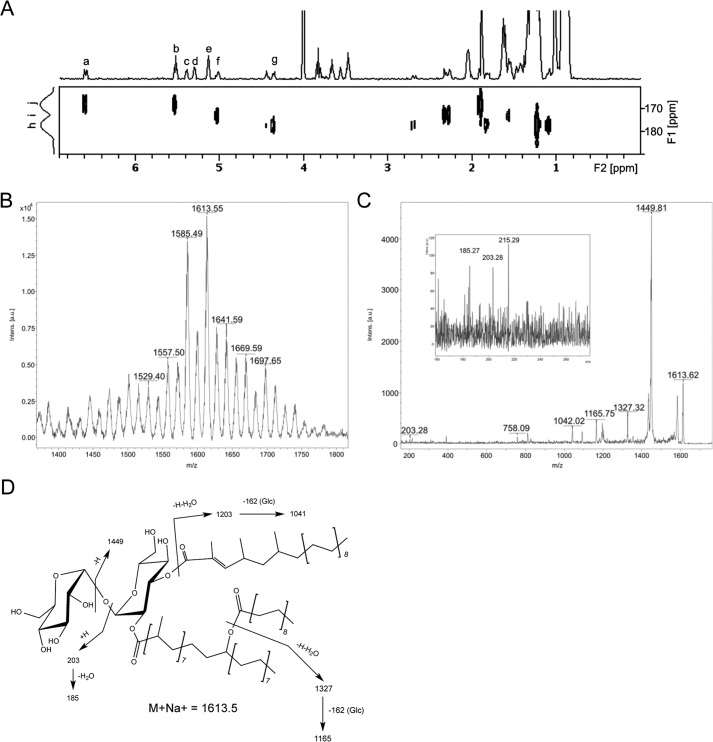
**Structural characterization of compound AT-X.** *A*, two-dimensional ^1^H-^13^C HMBC NMR spectrum of AT-X presenting coupling resonances to the carbonyl group region. The assignments of the various signals are reported in Table 1. *B*, the MALDI-TOF spectrum of AT-X. At this mass range, the individual isotope peaks are merged; hence, the mass number refers to the average (not monoisotopic) mass, and the mass inaccuracy is plus or minus an atomic mass unit or so. The masses are consistent with the sodiated molecular ion adduct for trehalose with three related fatty acyl groups as described under “Results.” *C*, the MS/MS spectrum of *m*/*z* 1613.5. All ions are sodiated. Masses are average, and the accuracy is as in *B. D*, a structure of AT-X consistent with the NMR and MS/MS data. A rationalization of the MS/MS data is shown. All ions are sodiated.

Noticeable in the *mmpL10* and *chp2* deletion mutants was the appearance of a novel [1-^14^C]propionate-labeled compound (AT-X) at the cell surface of the cells (Fig. 3*A*). This compound was not detected in cell pellet lipids and was not found in the wild-type *M. tuberculosis* strain or in the complemented *mmpL10* and *chp2* mutants. AT-X was prepared in non-radioactive form and analyzed for its constituent fatty acyl groups by formation of the corresponding fatty acyl methyl esters, trimethylsilyl ethers. The molecular or M-15 ions formed during GC/MS analysis of these compounds revealed the presence of saturated C16 and C18 fatty acyl groups (ratio of 1:2); C-25, -26, and -27 unsaturated fatty acyl groups presumed from precedent to be mycolipenoyl groups (ratio of 1:0.3:2.7); and monohydroxy C-34, 37, 40, 43, and 47 fatty acyl groups, presumed from precedent to be hydroxyphthioceranoyl groups, in a ratio of 2:1:14:2:4. The location of the hydroxyl group in the hydroxyphthioceranoyl groups was both 16 and 18 carbons from the end of the chain, as shown by fragment ions at *m*/*z* 313 and 341. MALDI analysis of purified AT-X revealed series of [M + Na^+^] ions separated by 14 units with the major peak at *m*/*z* = 1613.6 (isotope-averaged mass) (Fig. 8*B*). This corresponded to a triacylated trehalose esterified with stearoyl, C27 mycolipenoyl, and C43 hydroxyphthioceranoyl residues; the high and lower molecular weight ions are readily interpreted using other combinations of the fatty acyl groups. The NMR spectrum of AT-X (Fig. 8*A* and Table 1) showed that the structure of this lipid corresponds to a trehalose esterified on only one of the glucosyl units (system II; Table 1) with fatty acyl groups in positions 2 and 3. Most importantly, no downfield shift of any additional ring protons on either glucosyl residue was found, suggesting the presence of one acyl on the hydroxyl group of the hydroxyphthioceranoyl residue (Table 1). HMBC NMR analysis allowed the carbonyl groups of the three different fatty acyl chains to be identified due to differences in the hydrogens of the α and β carbons. In particular, the carbonyl of the mycolipenic acid was clearly coupled to the vinyl proton on C-3 of the fatty acyl group and then to H-3 of the system 2 glucosyl residue (Table 1). It then must follow that the stearoyl chain is attached to the hydroxyl group of the hydroxyphthioceranoyl residue, which is in turn attached to O-2 of the system 2 glucosyl residue. The MS/MS spectrum (Fig. 8*C*) of the sodiated ion at *m*/*z* = 1613.6 yielded fragment ions corresponding to the loss of an unsubstituted glucosyl residue (*m*/*z* = 1449) and release of sodiated unsubstituted glucose residue (*m*/*z* = 203 and the ion corresponding to the loss of water from it at *m*/*z* = 185), indicating that all of the fatty acyl groups are in one glucosyl residue. Other fragments consistent with this arrangement are shown in Fig. 8*C* as interpreted in Fig. 8*D*. This led us to propose the structure of AT-X shown in Fig. 8*D*.

**TABLE 1:** **Diagnostic ^1^H and ^13^C NMR chemical shifts of AT-X measured at 295 K in CDCl_3_-CD_3_OD (8:2, v/v)** Assignments were made using two-dimensional ^1^H-^1^H COSY and ^1^H-^13^C HSQC NMR spectroscopy. The superscript letters refer to resonances shown in Fig. 8*A*.

	Chemical shift (ppm)						
	System I		System II		Esterified fatty acyl OH	C=O chemical shift	C=O_3_ bond connectivity
	^1^H	^13^C	^1^H	^13^C			
H_1_/C_1_	5.138^e^	93.97	5.304^d^	91.07			
H_2_	3.499		5.036^f^				
H_3_	3.783		5.526^b^				
H_4_	3.419		3.631				
H_5_	3.619		4.199				
H_6_	3.706		3.752				
H_6′_	3.817		3.861				
CH_2_C**H(**OAcyl)CH_2_ (hydroxyphthioceranoyl)					4.371^g^		
C=O (stearoyl)						177.79	1.829; 2.698; 4.371^g^
C=O (hydroxyphthioceranoyl)						173.37	1.573; 5.036^f^
C=O (mycolipenoyl)						168.28	6.598^a^; 5.526^b^

Thus, for the first time, structural analyses revealed the existence in *M. tuberculosis* of an unsulfated acyltrehalose displaying mixed characteristics of SL and DAT/PAT with the unusual characteristic of a fatty acyl group esterified to the OH of the hydroxyphthioceranoyl residue. Its export to the cell surface could suggest that its formation is a response of the *mmpL10* and *chp2* mutants to the significant and potentially toxic build-up of DAT in the plasma membrane. Its finding at the cell surface of the *mmpL10* knock-out mutant further indicates that its translocation is independent from MmpL10. Whether its export proceeds through the SL translocation machinery remains to be determined but may be envisaged given the presence in AT-X of a hydroxyphthioceranyl chain esterifying the trehalose, similar to the situation in SL-I precursors. Analysis of the surface-exposed lipids extracted from [1-^14^C]propionate-labeled cultures of *Mtb*Δ*mmpL10* either treated with THL or untreated showed a significant and THL concentration-dependent decrease in AT-X production in the treated cells (78 and 91% inhibition after 24 h of exposure to 10 and 40 μg/ml of the compound, respectively), indicating that the acyltransferase(s) responsible for the formation of this acyltrehalose is susceptible to the effect of THL (Fig. 6*B*).

Consistent with earlier findings that the loss of production of DAT and PAT does not result in significant changes in the nature or abundance of related acyltrehaloses (28, 32), no changes in SL-I or any other known SL precursors were otherwise observed in any of the three DAT/PAT deletion mutants (data not shown).

## DISCUSSION

**FIGURE 9.**
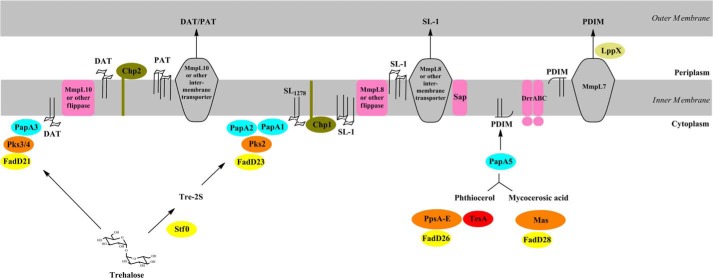
**Proposed DAT/PAT, sulfolipid, and phthiocerol dimycocerosate biosynthetic pathways.** *Left*, DAT and PAT biosynthetic pathway. The acyltransferase PapA3 initiates DAT and PAT biosynthesis on the cytosolic face of the plasma membrane by transferring a palmitoyl group to the 2-position of one of the glucosyl residues of trehalose to form trehalose 2-palmitate. PapA3 next transfers a mycolipenoyl group, synthesized by the polyketide synthase Pks3/4, to the 3-position of trehalose 2-palmitate to yield DAT. FadD21 is the fatty acyl AMP ligase that provides the activated fatty acyl starter unit to Pks3/4. DAT is then flipped across the plasma membrane either by an as yet unknown flippase or by MmpL10 and further elaborated with mycosanoyl, mycolipenoyl, and/or mycolipanolyl chains by Chp2 on the periplasmic face of the plasma membrane to form the penta-acylated PAT. DAT serves both as the donor and acceptor substrate in these Chp2-mediated transesterification reactions. DAT and possibly PAT are taken up by MmpL10 and/or by other as yet unknown periplasmic and outer membrane proteins from the outer leaflet of the plasma membrane and exported to the cell surface. The enzymes and transporters involved in the elongation, assembly, and export of sulfolipids (*middle*) and PDIM (*right*) and their localization in the bacterium are represented (for a recent review, see Ref. 2). PpsA-E is a type 1 polyketide synthase responsible for the formation of the phthiocerol; Mas is mycocerosic acid synthase; TesA is a type II thioesterase thought to be involved in the release of phthiocerol from PpsE; PapA5 is an acyltransferase responsible for the transfer of mycocerosic acids to phthiocerol to form PDIM; FadD23, FadD26, and FadD28 are long-chain fatty acyl-AMP ligases; Stf0 is a sulfotransferase; and PapA2 and PapA1 are acyltransferases responsible for the transfer of the first (palmitoyl or stearyl) and second ((hydroxy)phthioceranoyl) acyl chains, respectively, onto trehalose 2-sulfate to form the diacylated sulfolipid, SL_1278_. MmpL8 participates in the export of SL-I to the cell surface. MmpL7 participates in the export of PDIM. DrrABC and LppX are an ABC transporter and a periplasmic lipoprotein, respectively, required for PDIM to reach the cell surface. Sap is an integral membrane protein thought to facilitate the translocation of SL-I to the cell surface. The precise extent of sulfolipid and PDIM translocation mediated by MmpL7, MmpL8, Sap, LppX, and DrrABC has not yet been defined. Note that in the case of both sulfolipids and PDIM, the biosynthetic end products are formed on the cytoplasmic side of the plasma membrane prior to export to the periplasm and outer membrane, whereas the Chp2-mediated elaboration of PAT from DAT occurs on the periplasmic side of the membrane.

Altogether, the results presented herein are consistent with the DAT and PAT biosynthetic model presented in Fig. 9. DAT is formed in the cytosol upon sequential acylation of trehalose with a palmitoyl or stearoyl group and a fatty acyl product of Pks3/4 by PapA3 (32). DAT is then flipped across the plasma membrane either by MmpL10 or by an as yet unknown flippase and further elaborated with mycosanoyl, mycolipenoyl, and/or mycolipanolyl chains through Chp2-mediated trans-esterification reactions between DAT substrates on the periplasmic face of the plasma membrane to yield the penta-acylated PAT. Such a trans-esterification mechanism has precedent in *M. tuberculosis* and, in fact, seems to be a recurring theme in the biosynthesis of mycobacterial acyltrehaloses. Indeed, the three major mycoloyltransferases of *M. tuberculosis* known as the antigens 85A, 85B, and 85C catalyze the formation of trehalose dimycolate between two molecules of trehalose monomycolate on the periplasmic face of the plasma membrane (47). Likewise, Chp1 catalyzes trans-esterification reactions between two diacylated sulfolipid precursors (SL_1278_) on the cytoplasmic face of the membrane to yield SL-I, the final product of the sulfolipid biosynthetic pathway (33) (Fig. 9). DAT and possibly PAT are taken up by MmpL10 and/or by other as yet unknown periplasmic and outer membrane proteins from the outer leaflet of the plasma membrane and exported to the cell surface. The fact that PAT is synthesized on the periplasmic side of the plasma membrane calls into question the extent of (glyco)lipid translocation mediated by MmpL proteins. The localization of MmpL proteins in the plasma membrane could indeed suggest an involvement of these transporters in the translocation of (glyco)lipids either across the plasma membrane (“flippase” activity) or from the outer leaflet of the plasma membrane to the periplasm or outer membrane (intermembrane transport) or in both processes. The involvement of MmpL10 in the flipping of DAT across the plasma membrane would be consistent with the inability of the *mmpL10* null mutant to synthesize PAT. Alternatively, MmpL10 may mediate the intermembrane translocation of DAT (and possibly PAT), and the absence of PAT in the *mmpL10* knock-out be due to the failure of Chp2 to elaborate PAT in the absence of a functional MmpL10 protein, similar to the situation reported earlier for the sulfolipid biosynthetic pathway, where the elaboration of the fully acylated SL-I by Chp1 is potentiated by the presence of the MmpL8 transporter (33). In the case of an intermembrane transport, MmpL10 and possibly other mycobacterial MmpL proteins would take up their substrates from the outer leaflet of the plasma membrane and therefore resemble the classical Gram-negative RND transporters, which are known to pump out substrates from the periplasm rather than across the plasma membrane (48). An important correlate of this scenario is that, similar to Gram-negative RND transporters (49), MmpLs are likely to require the assistance of “flippases” and, possibly, additional periplasmic adapters, lipoproteins, and/or outer membrane proteins to deliver their substrates to or in the vicinity of the outer membrane. That MmpL-dependent translocation machineries involve such additional components is in fact already supported by a number of studies on the export of sulfolipids (33) (Fig. 9), phthiocerol dimycocerosates (50, 51) (Fig. 9), glycopeptidolipids (52, 53), and siderophores (54). Clearly, the precise definition of the compositions and export mechanisms of these MmpL-dependent translocation machineries await further investigations.

Interestingly, the formation of AT-X under conditions where DAT builds up in the plasma membrane highlights for the first time the existence of a cross-talk between the SL and DAT/PAT biosynthetic pathways. Independent from their interest in deciphering the biogenesis of unsulfated acyltrehaloses in *M. tuberculosis*, the set of recombinant strains described in this study, including those deficient in DAT and PAT translocation to the cell surface and those accumulating DAT in addition to a newly described acyltrehalose (AT-X), provide new opportunities for future studies aimed at understanding the role of these glycolipids in *M. tuberculosis* pathogenesis.

## Footnotes

3 J. M. Belardinelli and M. Jackson, manuscript in preparation.

## Footnotes

2 The abbreviations used are:

SL: sulfolipid(s)
DAT: 2,3-diacyltrehalose(s)
PAT: penta-acyltrehaloses
PDIM: phthiocerol dimycocerosates
THL: tetrahydrolipstatin.
